# The Critical Value of Maternal and Child Health (MCH) to Graduate Training in Public Health: A Framework to Guide Education, Research and Practice

**DOI:** 10.1007/s10995-022-03401-w

**Published:** 2022-05-07

**Authors:** Julianna Deardorff, Michelle Menser Tissue, Patricia Elliott, Arden Handler, Cheryl Vamos, Zobeida Bonilla, Renee Turchi, Cecilia Sem Obeng, Jihong Liu, Holly Grason

**Affiliations:** 1 School of Public Health, University of California, Berkeley, CA USA; 2 Health Resources and Services Administration, Maternal and Child Health Bureau, Rockville, MD USA; 3 School of Public Health, Boston University, Boston, MA USA; 4 School of Public Health, University of Illinois, Chicago, IL USA; 5 College of Public Health, University of South Florida, Tampa, FL USA; 6 School of Public Health, University of Minnesota, Minneapolis, MN USA; 7 School of Public Health, Drexel University, Philadelphia, PA USA; 8 School of Public Health, Indiana University, Bloomington, IN USA; 9 School of Public Health, University of South Carolina, Columbia, SC USA; 10 School of Public Health, Johns Hopkins University, Baltimore, MD USA; 11 Community Health Sciences Division, School of Public Health, University of California, 2121 Berkeley Way West, 94720-7360 Berkeley, CA USA

**Keywords:** Maternal and child health, Training, Education, Life course, Research, Practice, CEPH

## Abstract

**Introduction:**

In light of persistent health inequities, this commentary describes the critical role of maternal and child health (MCH) graduate training in schools and programs of public health (SPPH) and illustrates linkages between key components of MCH pedagogy and practice to 2021 CEPH competencies.

**Methods:**

In 2018, a small working group of faculty from the HRSA/MCHB-funded Centers of Excellence (COEs) was convened to define the unique contributions of MCH to SPPH and to develop a framework using an iterative and consensus-driven process. The working group met 5 times and feedback was integrated from the broader faculty across the 13 COEs. The framework was further revised based on input from the MCHB/HRSA-funded MCH Public Health Catalyst Programs and was presented to senior MCHB leaders in October 2019.

**Results:**

We developed a framework that underscores the critical value of MCH to graduate training in public health and the alignment of core MCH training components with CEPH competencies, which are required of all SPPH for accreditation. This framework illustrates MCH contributions in education, research and evaluation, and practice, and underscores their collective foundation in the life course approach.

**Conclusions:**

This new framework aims to enhance training for the next generation of public health leaders. It is intended to guide new, emerging, and expanding SPPH that may currently offer little or no MCH content. The framework invites further iteration, adaptation and customization to the range of diverse and emerging public health programs across the nation.

The United States faces long-standing and persistent challenges in maternal and child health (MCH), particularly regarding infant and maternal mortality, that have negative repercussions across the lifespan for individuals, families, and communities (Holdt Somer, Sinkey, & Bryant, 2017; Leonard, Main, Scott, Profit, & Carmichael, 2019). Historically marginalized groups disproportionately bear the burden of these challenges (Martin, Hamilton, Osterman, & Driscoll, 2019). The racial reckoning of 2020 and the racially disparate health impacts of the coronavirus disease 2019 (COVID-19) pandemic underscore and augment existing inequities, illuminating the role of structural issues that bolster them (Gur et al., 2020; Nuru-Jeter et al., 2018). At this moment in history, Schools and Programs of Public Health (SPPH) have an important responsibility to update their curricula and training opportunities to better address health disparities and share strategies to guide curriculum development and practice. Graduate programs in MCH, with their established history of approaching public health from community-engaged, systems-oriented, interdisciplinary, and life course perspectives, are uniquely positioned to train the next generation of leaders to promote health equity, develop and assess responsive community-based interventions, and challenge structures and policies that sustain inequities.

In 2016, the Council on Education for Public Health (CEPH) substantially revised guidelines for accreditation with a focus on innovation, leadership, and practice-based knowledge, skills, and competencies (Council on Education for Public Health, October 2016). At that time, MCH training within SPPH already championed many of these competencies, with MCH faculty providing integrated, cross-professional, community-based training and leadership courses that cut across disciplines and divisions within schools. As such, SPPH with distinct MCH programs ultimately benefit by having faculty poised to support larger educational initiatives with important lessons learned from the MCH field that support all disciplines in public health. Here, we present a framework that highlights the critical value of graduate training in MCH within SPPH and illustrate the alignment of the framework with CEPH competencies.

## Background - MCH Education and Training

National acknowledgment of the need for MCH training can be traced back to the original authorization of Title V of the Social Security Act, which was signed into law in 1935 and allocated funds to states to “*extend and improve”* health and welfare services for mothers and children, including through short courses and continuing education (Social Security Administration, n.d.-a). In 1947, the federal government funded four universities to enhance administration of state-based programs. Now, administered by the Health Resources and Services Administration’s (HRSA) Maternal and Child Health Bureau (MCHB), the MCH Training Program portfolio includes 13 *Centers of Excellence in MCH Education, Science and Practice* (COEs) and 9 *MCH Public Health Catalyst Programs* (MCH Workforce Development Programs, n.d.). These programs establish and maintain academic-practice partnerships with MCH with state and local health departments (Title V programs) and related MCH organizations to ensure learning and promote translation of research and evaluation to practice and policy.

Although the 22 COE and Catalyst programs work collectively to advance the education and training of the MCH workforce, they represent a small number of schools and programs across the nation. In fact, the number of SPPH increased markedly in the U.S. from 1992 to 2016, with a 300% increase in the number of public health degrees conferred during this period (Leider, Plepys, Castrucci, Burke, & Blakely, 2018) and many new programs and partnerships emerging at Hispanic serving institutions and historically Black colleges and universities. This uptick in the number of programs – along with a surge in student interest in public health since the pandemic – demands broader dissemination of the co-learning and advancements that have taken place in MCH public health graduate education programs to strengthen SPPH curricula more broadly and further support the training of the public health workforce.

The framework presented here offers a guide to encourage other graduate programs to pursue MCH as a focal point in their curricula. We posit that all disciplines in SPPHs can benefit from the MCH focus on the life course, community health, and structural and social determinants of health to successfully address CEPH competencies. In particular, the framework provides a guide to SPPH that are in the process of developing curricula in MCH.

### Development of the Framework

**Fig. 1:**
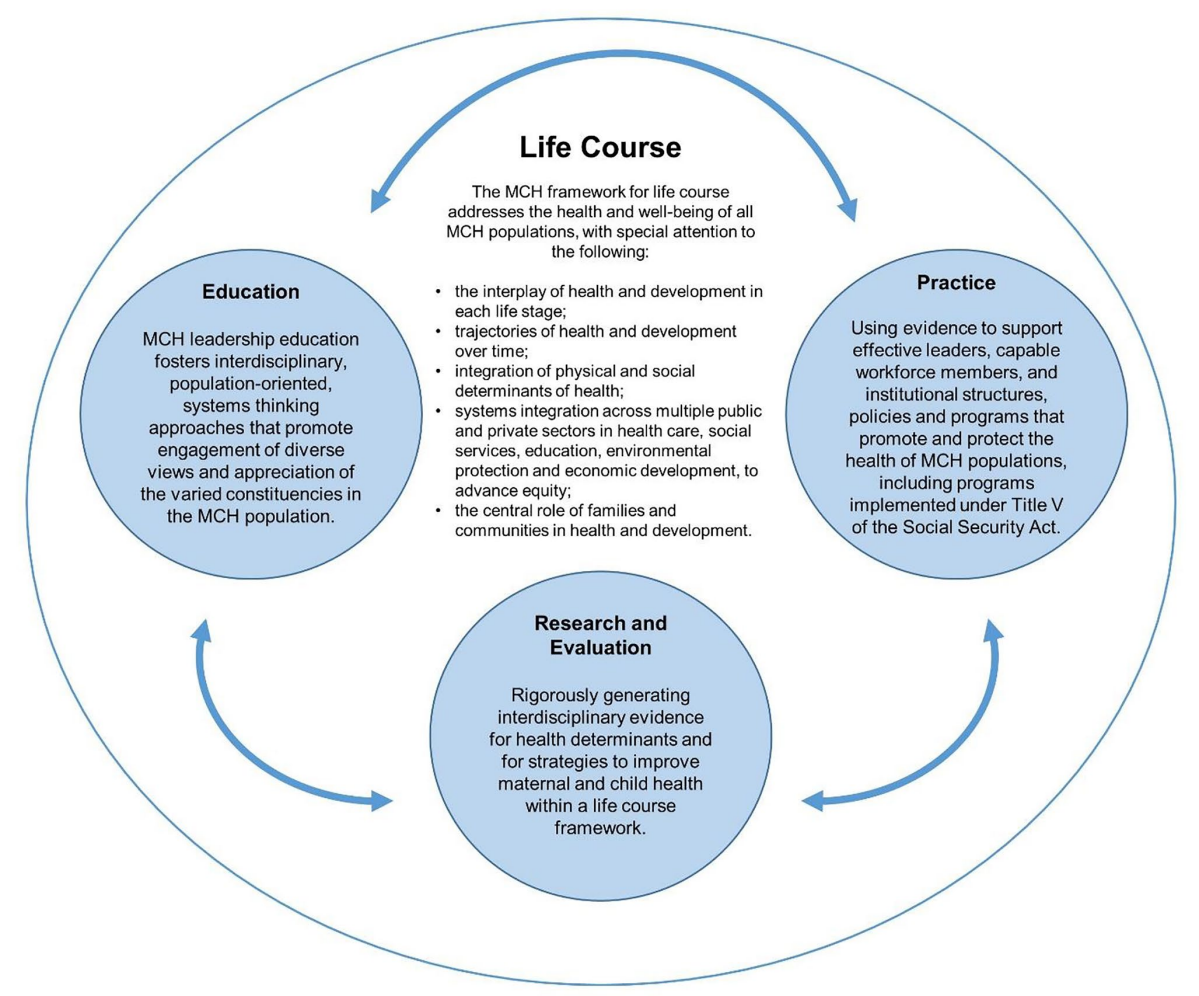
The Critical Value of Maternal and Child Health (MCH) to Graduate Training in Public Health: A Framework to Guide Education, Research and Practice

In March 2018, faculty from the COEs explored how to best describe the value of MCH training in public health graduate education. A working group met 5 times (June 2018 - February 2019) to identify core elements that define the unique contributions of MCH to SPPH using an iterative and consensus-driven process. To inform initial themes, the 13 COE programs participated in a discussion to answer the question “How are you (MCH) unique within your school of public health?” This process prompted the development of a framework (Fig. 1), which outlines the core components of public health MCH graduate training for education, research and evaluation, and practice and illustrates the interplay among the components. The framework was presented to COE and Catalyst Programs and feedback was incorporated. Then, the framework was presented to MCHB leadership in October 2019 for consideration and potential use. Here, we present each component of the framework – (1) Life Course, (2) Education, (3) Research and Evaluation, (4) Practice – and highlight how SPPH benefit from MCH graduate training. Table 1 illustrates the alignment between the core components of the framework and the 2021 CEPH competencies, and provides sample course titles for each domain.

**Table 1:** Crosswalk of Critical Value of MCH Graduate Training in Public Health Framework, Sample Course Titles, and 2021 CEPH Foundational Competencies

Critical Value of MCH Graduate Training in Public Health Framework	Sample Course Titles from MCHB-funded Centers of Excellence and Catalyst Programs*	CEPH Foundational Competencies (2021)
Life Course	Life Course Perspectives on Health Children with Special Health Care Needs: A Systems Perspective Foundations in Maternal and Child Health	Profession and Science of Public Health Factors Related to Human Health Public Health and Health Care Systems
Education	MCH Collaborative Leadership Seminar Reproductive Health, Rights, and Justice Children’s Health, Life Course and Equity Perspectives Embodying Gender: Public Health, Biology, and the Body Politic	Factors Related to Human Health Leadership Communication Inter-professional and/or Inter-sectoral Practice Systems Thinking
Research and Evaluation	Secondary Data Analysis in Maternal and Child Health Reproductive and Perinatal Epidemiology Using Data to Design and Evaluate Family Planning Programs and Policies	Evidence-based Approaches to Public Health Inter-professional and/or Inter-sectoral Practice
Practice	Maternal and Child Health Policy and Advocacy Translating Evidence for Maternal and Child Health Practice	Profession and Science of Public Health Evidence-based Approaches to Public Health Planning and Management to Promote Health Policy in Public Health

*For a full list of MCHB-funded Centers of Excellence and Catalyst Programs visit: https://mchb.hrsa.gov/programs-impact/focus-areas/building-mch-leaders-mch-workforce

### Life Course

The life course perspective is central to the framework and anchors MCH education, research and evaluation, and practice. A life course approach addresses the health and wellbeing of MCH populations with special attention to health and development at each life stage, and over time, and integrates the central role of families and communities. Physical as well as social determinants are considered, and there is a keen focus on equity through the integration of interdisciplinary and inter-professional partnerships across public and private sectors. Within our working group, a consensus emerged that although some public health disciplines address limited aspects of the life course in their training for graduate students, the life course perspective embedded in this framework is fundamental in MCH. There was broad agreement across COE and Catalyst programs that faculty in MCH play a central role in providing life course education and mentoring not just to MCH students but to students and faculty across disciplines and divisions within SPPH.

Integrating the life course perspective into all aspects of graduate training (education, research and evaluation, and practice) meets key CEPH competencies, including those focused on factors related to human health. As detailed in the *Handbook of Life Course Health Development* (2018), decades of research have underscored the essential importance of a life course approach to health as key to understanding phenomena, such as the role of the intrauterine environment in adult chronic disease, the interplay of biologic, social and environmental determinants of health and epigenetics, the role of government, policy and economic structures in access to services, and generations of inequities resulting from racism, heterosexism, and ableism (Bailey, Feldman, & Bassett, 2021; Boyce, Levitt, Martinez, McEwen, & Shonkoff, 2021; Braveman, 2014; Halfon et al., 2018; Halfon, Larson, Lu, Tullis, & Russ, 2014; Lu, 2019). In their seminal 2014 article, Lu and Halfon built on the long history of life course research to explain racial inequities in birth outcomes, highlighting its utility to the field of MCH (Lu, 2014). The framework presented here, anchored by the life course perspective, reflects the approach needed to guide the interdisciplinary nature of evolving graduate program curricula that will prepare the growing public health workforce for persistent and new challenges.

### Education

MCH education in SPPH fosters interdisciplinary, population-oriented, and systems thinking approaches. Often, MCH courses are the primary exposure that public health graduate students have to life course topics including human development, intergenerational transmission of risk for disease, reproductive and perinatal epidemiology including epigenetics, and critical developmental windows of susceptibility and opportunity for health promotion. These factors are important to human health as outlined in CEPH requirements. High-quality MCH pedagogy also prepares students to understand inequities across the lifespan and the impact of racism on health at every developmental stage, both critical to educating leaders who are prepared to tackle the complex public health issues that the nation currently faces (Raskind et al., 2019). Moreover, MCH leadership competencies (Health Resources and Services Administration Maternal and Child Health Bureau, 2018), which also inform MCH curricula, emphasize cultural humility, inter-professional team building, and communication across sectors, providing a strong basis for education and leadership training to all graduate students to meet CEPH requirements in SPPH. Schools and programs with strong MCH public health content prepare students to work across systems and question racial and colonial legacies that are embedded in policies, communities, and systems of care. Moreover, MCH public health education reaches students and faculty from other disciplines – nursing, medicine, city planning, pharmacy, psychology, social welfare, business, public policy, and law – to promote inter-professional, experiential learning and leadership training (McHugh, Margolis, Rosenberg, & Humphreys, 2016), thereby addressing core CEPH competencies. SPPH can leverage the skills and knowledge base that are central to MCH to enhance and broaden the preparation of students across all disciplines and programs.

### Research and Evaluation

This component of the framework refers to generating robust interdisciplinary evidence for health determinants and strategies to improve health within a life course framework, which have been at the core of MCH training since its earliest days. MCH research and evaluation promotes strategies that integrate knowledge of the emergence of health problems over the course of human development, rigorous evaluation of interventions, and translation of this information into effective public health practice, policy, and systems-level change. Researchers and evaluators in MCH take both a *systems* and *individual* approach, routinely situating their findings with respect to the structural determinants of health, and examining the implications of their findings across levels of the social-ecological model and the entire lifespan (Braveman, 2014). MCH training in research and evaluation within SPPH promotes CEPH competencies in several ways. It takes account of key biologic and genetic factors across the life course related to human health. It promotes CEPH competencies around evidence-based approaches to public health and inter-professional/inter-sectoral and interdisciplinary approaches to research and evaluation.

As economic, scientific, and cultural forces transform health systems, well-trained researchers are needed who are skilled not only in examining complex challenges but developing and testing interventions at multiple levels. Maternal and child health research often has its origins in the experiences of MCH practitioners and the communities they serve. This research requires skills training inherent in MCH, including drawing on a variety of research methods including epidemiology, health services research, survey research, evaluation, policy analysis, econometrics, and innovative methods necessary to analyze large complex public health datasets, qualitative and participatory methods, and, more recently, big data. The life course and multilevel approaches of MCH research provide training in interdisciplinary methods that trainees require and further enrich the overall scholarship produced at SPPH.

### Practice

The practice component of the framework includes the use of evidence to support effective leaders, capable workforce members, and institutional structures, policies, and programs to promote and protect the health of all populations. MCH training in SPPH is rooted in community-engaged approaches, with a long history of academic-practice partnerships that facilitate the translation of research and evaluation to policy and practice (Reno, Warming, Zaugg, Marx, & Pies, 2021; Tiedje, 2005). A practice orientation, long championed in MCH, prepares students across disciplines in SPPH to apply theoretical frameworks, including life course perspective and social disparities lens, to the development of policies and programs that are tailored to state, local, and community contexts (Fraser, 2013; Kroelinger et al., 2014; Pies, Parthasarathy, & Posner, 2012). SPPH that support the delivery of MCH content and skills produce students equipped to use epidemiologic methods to describe public health problems in the unique context of communities; to interpret and apply data analyses for public health policy and practice; and to evaluate the impact of programs and policies on populations across the lifespan. MCH graduate training also emphasizes cultural responsiveness and promotes an understanding of how cultural practices and values influence the design and implementation of public health programs, further preparing graduate students in SPPH to enter the workforce. Through rigorous hands-on community-based training, engagement, and connections to practice, MCH educators establish and enhance networks among trainees and professionals in the field, forming essential interdisciplinary mentorship and training structures that benefit all students in SPPH broadly. In return, students provide valuable contributions to - and sustain academic-practice partnerships among - community partners and organizations.

Federal funding for MCH training in SPPH is linked inextricably to MCH practice through the Title V Block Grant legislation (Social Security Administration, n.d.-b), which specifically names training as a component of Title V’s discretionary funding, known as the Special Projects of Regional and National Significance (SPRANS). As a result, MCH training programs in SPPH maintain close ties with Title V state and local MCH practice partners and collaborate to develop and implement interventions across life course domains (Health Resources and Services Administration, n.d.-b). Through these partnerships, students across all disciplines in SPPH can obtain premier field placements for applied practice and integrated learning experiences, as required by CEPH in public health graduate programs.

### Future Directions

Our goal is to highlight MCH in graduate public health education by illustrating how MCH content and praxis (as presented in our framework) facilitate the achievement of CEPH competencies. Each SPPH is built on the pillars of education, research and evaluation, and practice. Schools and programs can ensure that each of these pillars is grounded in the tenets of life course and strengthened by specific MCH content. Expanding MCH pedagogy and training in SPPH is aligned with the work more broadly to update graduate public health curricula nationwide. For SPPH to effectively train professionals and scholars of the future, there needs to be a strong, diverse, and visible MCH faculty to conduct and translate research on the cutting-edge issues that directly affect communities, and to serve as key academic partners who can support MCH practice, including state Title V agencies. Well-established MCH training programs, like those with Catalyst or COE funding, serve as models for the curricular expansion of MCH in SPPH that currently have little to no MCH content.

The framework presented here can be used as a communication tool with federal and academic leaders, including deans and chairs in SPPH, to highlight the unique contributions of MCH education in the broader public health landscape. The framework supports the many arguments that can be made to department chairs and deans about the benefits of MCH programs to all public health disciplines, particularly as SPPH continue to recognize the explicit need for robust anti-racist pedagogy and praxis and interdisciplinary approaches to tackle persistent health inequities. Although this framework provides a foundation to illustrate core areas where MCH is of critical value to SPPH and alignment with CEPH competencies, over time, the framework is expected to evolve as public health graduate training continues to be responsive to historical, political, economic, educational, and cultural contexts.

## Conclusion

The Maternal and Child Health Bureau recently released a strategic plan to guide the field over the next 10–15 years (Health Resources and Services Administration, 2021). The strategic plan elevates the importance of public health graduate education in MCH as a key MCH-specific workforce initiative. Further highlighting the need for MCH-specific workforce development is the growing number of students interested in graduate education in public health and an increasing number of graduate institutions with MCH coursework. The framework offers an actionable lens to view public health training through the fundamental interconnectedness of education, research and evaluation, and practice, grounded in the life course approach. It offers guiding principles for integrating MCH content and praxis into new and existing SPPH. The framework also supports the need for a diverse, competent, and committed workforce with a strong foundation in MCH to address health inequities across the lifespan and to support future public health leaders. MCH public health academe will benefit from ongoing self-reflection to ensure high standards as well as engagement with non-MCH colleagues to underscore and elevate the importance of graduate training in MCH to meet CEPH requirements and achieve broader public health goals.

## Data Availability

Not applicable.
